# The complete mitochondrial genome of Aesop slipper lobster *Scyllarides haanii* (De Haan, 1841)

**DOI:** 10.1080/23802359.2020.1823274

**Published:** 2020-09-22

**Authors:** Hongtao Liu, Guofu Wang

**Affiliations:** a Key Laboratory of Utilization and Conservation for Tropical Marine Bioresources, (Hainan Tropical Ocean University), Ministry of Education, Sanya, China; b Hainan Provincial Key Laboratory of Tropical Maricultural Technologies, Hainan Academy of Ocean and Fisheries Sciences, Haikou, China

**Keywords:** *Scyllarides haanii*, mitochondrial genome, phylogenetic analysis

## Abstract

The complete mitochondrial genome of *Scyllarides haanii* from South China Sea was first determined and characterized. With a length of 15,666 bp, the circular mitogenome of *S. haanii* consists of 22 tRNA genes, two rRNA genes, 13 protein-coding genes (PCGs), and one control region. The nucleotide composition is significantly biased (A, G, T, and C was 32.05%, 12.45%, 33.97%, and 21.53%, respectively) with A + T contents of 66.02%. Two PCGs used an unusual initiation codon, and five PCGs were ended with an uncomplete or abnormal stop codon. One microsatellite was identified in the mitogenome located in ND4 gene. Phylogenetic analysis demonstrated that *S. haanii* was first clustered with *Scyllarides squammosus*, then together with S*cyllarides latus*.

*Scyllarides haanii*, known as humpbacked slipper lobster, is a marine decapod crustacean in the family Scyllaridae. Its dorsal midline of the 2nd and 3rd segments strongly ridged and that of the 4th segment producing a remarkable hump (Holthuis 1991). *Scyllarides haanii* occurs near in rocky substrates in depth ranging from 10 to 135 m (Griffin and George 1973). It enjoys an wide distribution in Indo-West Pacific region: from the Red Sea and the western Indian Ocean to Japan, Korea, China, Indonesia, Australia and Hawaii (Raghu Prasad and Tampi 1968; Wardiatno et al. 2016). Due to its solitary and nocturnal habit, as a rule it is only incidentally caught for sale fresh at local markets. It is believed to be the largest of the Scyllarides species, its overexploitation has forced a limited fishing season, size restrictions and no berried females may be taken (Santana et al. 2007). In the past the study on *S. haanii* only its occurrence in the Hawaiian has been reported (Morin and MacDonald 1984).

The samples were collected from Qinglan fishing port of Wenchang, China (N19°31'22.83", E110°49'50.69"), and stored in the marine crustacean specimen room (C20190709SH) in Qionghai research base of Hainan Academy of Ocean and Fisheries Sciences for reference and DNA extraction.

The complete mitogenome sequence of *S. haanii* is 15,666 bp in length (GenBank Accession no. MN817127). Its base content was 32.05% A, 12.45% G, 33.97% T, and 21.53% C. The 66.02% of (A + T) showed great preference to AT. It contained 22 tRNA genes, two rRNA genes, 13 protein-coding genes (PCGs), and one control region (D-loop). Four PCGs (ND1, ND4L, ND4 and ND5), eight tRNA genes and two rRNA genes were encoded on the light strand, the others were encoded by the heavy strand.

The 22 tRNA genes of the *S. haanii* mitogenome rang in length from 63 bp to 73 bp. tRNA-Leu and tRNA-Ser have two copies each identified the different codon (tRNA-leu uses TAA and TAG; tRNA-Ser uses TCT and TGA).The 12S rRNA is located between tRNA-Val and D-loop with the length of 851 bp, and the 16S rRNA is 1405 bp, located between tRNA-Val and tRNA-Leu. Except for two PCGs using an abnormal start codon (ND1 use GTG; COX1 uses CAA), the others use a usual initiation codon ATT or ATG. We also found that except for eight PCGs using TAA or TAG, the stop codon of the other five genes were abnormal: COX1, COX2, ND4, and CYTB use a single base T––; ND5 uses AGG. With a length of 743 bp, the control region is located between 12S rRNA and tRNA-Ile. One microsatellite (SSR) was found in the mitogenome of *S. haanii* using MISA software. The (TA)_6_ SSR was located in the codon region of ND4 gene.

A phylogenetic tree was constructed based on the 13 PCGs nucleotide sequences of 19 species mitogenome to investigate the phylogenetic relationship of *S. haanii* in the Achelata using neighbor-joining (NJ) method with 1000 bootstrap replicates. The result (Figure 1) that *S. haanii* was first clustered with *Scyllarides squammosus*, then together with S*cyllarides latus*. This phylogenetic analysis was consistent with the previous work (Palero et al. 2016).

**Figure 1.**
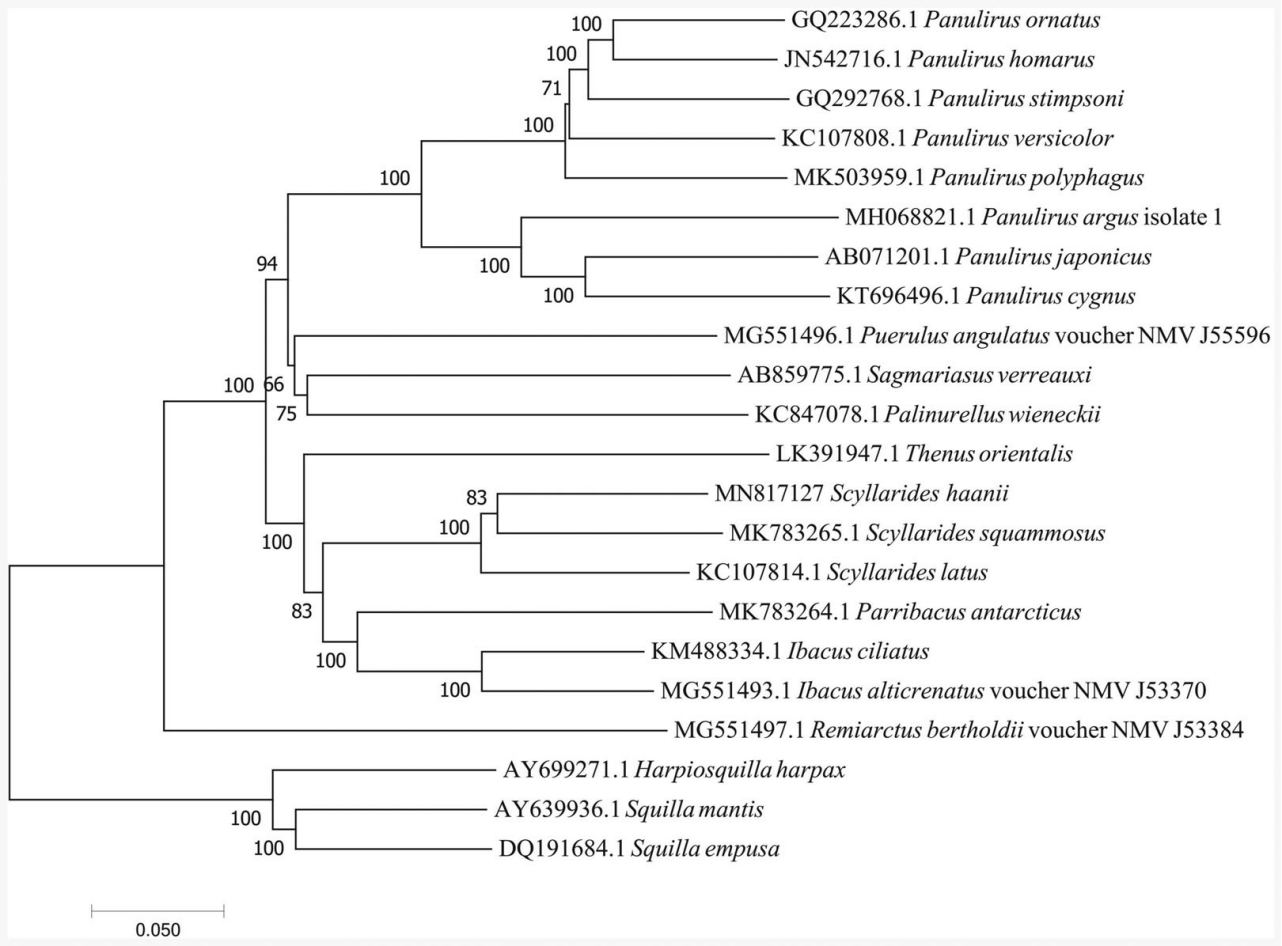
Phylogenetic tree of the complete mitogenome of 19 species in Achelata. *Harpiosquilla harpax*, *Squilla empuse* and *Squilla mantis* were used as outgroups.

## Data Availability

The data that support the findings of this study are available in “figshare” at https://doi.org/10.6084/m9.figshare.12805703.v1
